# Identification of histone modifications in biomedical text for supporting epigenomic research

**DOI:** 10.1186/1471-2105-10-S1-S28

**Published:** 2009-01-30

**Authors:** Corinna Kolářik, Roman Klinger, Martin Hofmann-Apitius

**Affiliations:** 1 Department of Bioinformatics, Fraunhofer Institute Algorithms and Scientific Computing (SCAI) Schloß Birlinghoven, D-53754 Sankt Augustin, Germany; 2 Department of Applied Life Science Informatics, Bonn-Aachen International Center for Information Technology (B-IT) Dahlmannstrasse 2, D-53113 Bonn, Germany

## Abstract

**Background:**

Posttranslational modifications of histones influence the structure of chromatine and in such a way take part in the regulation of gene expression. Certain histone modification patterns, distributed over the genome, are connected to cell as well as tissue differentiation and to the adaption of organisms to their environment. Abnormal changes instead influence the development of disease states like cancer. The regulation mechanisms for modifying histones and its functionalities are the subject of epigenomics investigation and are still not completely understood. Text provides a rich resource of knowledge on epigenomics and modifications of histones in particular. It contains information about experimental studies, the conditions used, and results. To our knowledge, no approach has been published so far for identifying histone modifications in text.

**Results:**

We have developed an approach for identifying histone modifications in biomedical literature with Conditional Random Fields (CRF) and for resolving the recognized histone modification term variants by term standardization. For the term identification *F*_1 _measures of 0.84 by 10-fold cross-validation on the training corpus and 0.81 on an independent test corpus have been obtained. The standardization enabled the correct transformation of 96% of the terms from training and 98% from test the corpus. Due to the lack of terminologies exhaustively covering specific histone modification types, we developed a histone modification term hierarchy for use in a semantic text retrieval system.

**Conclusion:**

The developed approach highly improves the retrieval of articles describing histone modifications. Since text contains context information about performed studies and experiments, the identification of histone modifications is the basis for supporting literature-based knowledge discovery and hypothesis generation to accelerate epigenomic research.

## Background

The expression of genes is regulated by their accessibility for the transcription machinery, which is controlled by the chromatine structure. Histones, structure forming proteins of chromatin, play an important role in the building of a closed or open chromatin state. Key players are several chemical groups, small molecules or processes modifying amino acids at histone tails. They change the physico-chemical properties of the amino acids and mark histone proteins for the recruting of other proteins participating in the formation of the different chromatin structure states [1]. Known histone modifying groups, molecules or amino acids modified by transformation processes are given in Table 1. More than 70 sites for histone post-translational modifications (PTMs) have been reported [2].

**Table 1:** Histone modifications. Histone modifying groups, molecules, and processes [1,41-43].

Modification Types	Modification examples
Groups	acetyl, methyl, phosphate, ADP ribosyl, carbonyl, sumoyl
Molecules	biotin, ubiquitin
Process	proline isomerization, arginine deamination (citrulline generation)

Combinations of histone modifications on one or different histones form a kind of histone code and carry different functionalities [3]. Until now the histone code and its functional relations are not fully understood and not all factors taking part in the regulation of the modifications itself are known.

One factor influencing histone modifications is the environment of organisms, like the nutritional deprivation, chemical toxins, xenobiotics, and drugs as well as psychosocial exposure during early developmental stages. External and intrinsic substances affect histone modifying enzymes which can lead to a heritable change in histone modification patterns. This alters the expression of a number of genes, which is in general a property of organisms to adapt to varying environmental conditions by forming environment-dependent phenotypes without changing the genetic code [4-6]. However, studies show that abnormal changes in modification patterns could be the basis or an additional factor for the development of cancer, mental disorders or late onset diseases, e.g. diabetes type II and cardiovascular medical conditions [7,8]. In addition to DNA methylation, PTMs of histone proteins are one of the major components studied in epigenomics. Information about histone binding proteins, genes concerned, studied cell and tissue types of different organisms, and chemical substances being related with certain histone modification patterns are needed for discovering the modification functionality and influence on diseases. 

Resources containing information about histone modifications are scientific articles and databases. The UCSC Genome Browser [9] is a common resource for genomic data usable for annotation. ChromatinDB [10] is a database of genome-wide histone modification patterns of *Saccharomyces cerevisiae*. The Histone Database [11] contains histone sequences of many organisms and their alignments as well as amino acid specific histone modifications, whereas they are not linked to phenotypes, cell or tissue types or experimental conditions. In its current version, ChromatinDB and Genome Browser do not support the analysis of histone modifications across species, related to various cell or tissue types or certain diseases. A resource providing context information related to histone modifications is the collection of abstracts of scientific publications in public text repositories like PubMed [12]. It covers the conditions of performed experiments and studies, like cell types or diseases which are not in the focus of the existing repositories and hence are not represented there. An automated support for exploring epigenomic texts would allow for finding new hypotheses on effects of histone modifications, its genomic positions, altering chemical substances, the developed phenotypes or diseases, cell and tissue types, the influenced expression state of a gene and the studied organism, especially if researchers are working in distinct fields. Smalheiser [13] and Hristovski [14] have shown that the automated analysis of terms or concepts linking two disparate text corpora can support the discovery of implicit relations between two subjects.

**Figure 1:**
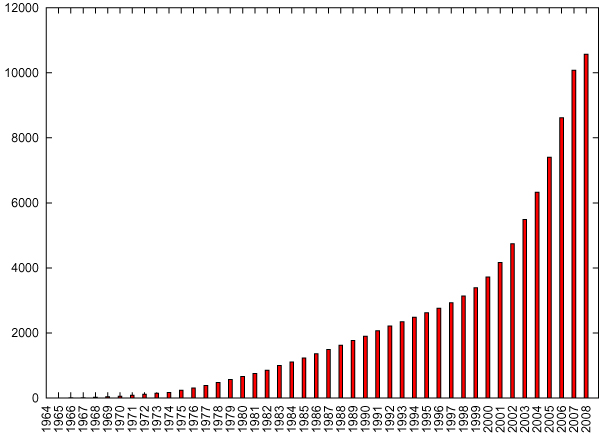
**Statistics on articles published about histone modifications in PubMed**. Number of published articles about histone modifications in MEDLINE obtained by a coocurrence search of histone terms and modification terms with ProMiner.

In July 2008, PubMed contained over 24,600 abstracts dealing with epigenomics (PubMed search using the term '*epigenetics*'). About half of them talk about histone modifications. Since methods have been ready for experiments at high throughput rate (e.g. ChIP-chip) and the influence of epigenomical factors onto disease states, cell differentiation, etc. received increased awareness, the article number has been growing and is expected to grow in the future (cf. Figure 1). On average, over 1000 articles have been published every month in the last two years. Therefore, automated methods need to be applied for supporting semantic text retrieval as well as for extracting histone modification related information. A fundamental step complying with this demand is the identification of histone modification mentions in text.

Several approaches have been developed for recognizing biomedical named entities, like proteins, genes, Single Nucleotide Polymorphisms, chemical substances, and diseases [15-18]. They are based on machine learning methods, rules, dictionaries or are combinations of them. To support epigenomic research the literature database PubMeth [19] has been recently set up which is based on text mining focussing on DNA methylation and cancer.

To the best of our knowledge no approaches have been published so far dealing with the identification of histone modification mentions in text and its application.

### Nomenclature and terminology used for histone modifications

A nomenclature for histone modifications was devised at the first meeting of the Epigenome Network of Excellence [20] in 2004 [21] and was first published as the Brno nomenclature by Turner [22] in 2005. Nevertheless, the way how histone modifications are described in text is not uniform and the usage of the official nomenclature is not common, which makes its identification difficult. This is a widespread habit also observable for the use of nomenclatures of other biomedical entities, like Single Nucleotide Polymorphisms (SNPs) or the use of the HUGO nomenclature for genes [23,24]. The following examples are typical mentions of histone modifications as they can be found in scientific text:

• H3K9me3; (41),

• Me3-K9 H3; (1),

• Me(3)-K9 H3; (78),

• H3K9 tri-methylation; (7),

• H3-K9 trimethylation; (28),

• H3 Lys9 trimethylation; (11),

• H3 tri-methylated at lysine 9; (14),

• histone H3 trimethylated at lysine (K) 9; (3),

• K9 trimethylation at histone H3; (36),

• K9-trimethylated histone H3; (15),

• tri-methylation of H3 at lysine residues K9; (0),

• trimethylated H3K9; (18).

The numbers in brackets provide the quantity of abstracts obtained with a PubMed search. Only the term '*H3K9me3*' corresponds to the Brno nomenclature. H3 stands for the protein '*histone 3*', the letter '*K*' specifies the amino acid lysine and '*9*' its position within the protein sequence. Furthermore, words starting with '*trimethyl*' and '*me3*' explain that the lysine carries three methyl groups as chemical modification.

The examples given above show, that a simple search strategy or a dictionary based approach is not able to find all description variants related to a certain histone and modification type. We apply Conditional Random Fields (CRFs) for recognizing histone modification descriptions in text.

To resolve the term variant problem and improving text retrieval we developed rules for mapping the identified terms to a standard form corresponding to the Brno nomenclature. Furthermore, a histone modification term hierarchy was built for organizing the standardized terms and enabling semantic text search.

## Methods

We developed an approach which aims at identifying histone modifications in text with CRF and resolving term variants by transforming them into terms corresponding to the Brno nomenclature. In a following step they are normalized and mapped to a generated histone modification term hierarchy.

### Conditional Random Fields

Conditional Random Fields [25-27] are a probabilistic model for computing the probability P ($\vec{y}$|$\vec{x}$) of a possible label sequence $\vec{y}$ = (*y*_1_,..., *y*_*n*_) given the input sequence $\vec{x}$ = (*x*_1_,..., *x*_*n*_), which is also called the observation sequence. In the context of Named Entity Recognition it corresponds to the tokenized text. This is the sequence of tokens which is obtained by a process which splits text at white space, punctuation marks and parentheses in general.

The label sequence is coded using the label alphabet ℒ = {*I-Hmod*, *O*, *B-Hmod*} where *y*_*i *_= *O *means that *x*_*i *_is not an entity of interest, *y*_*i *_= *B-Hmod *means that *x*_*i *_is the beginning of a histone modification mention and *y*_*i *_= *I-Hmod *means that *x*_*i *_is the continuation of it. A linear-chain CRF is an undirected probabilistic graphical model

$$P_{\vec{\lambda }}(\vec{y}|\vec{x})=\frac{1}{Z(\vec{x})}\cdot \prod_{j=1}^{n}{\text{e}}^{\left(\sum_{j=1}^{n}\sum_{i=1}^{m}\lambda_{i}f_{i}\left(y_{j-1},y_{j},\vec{x},j\right)\right)}.$$

with normalization to [0, 1] given by

$$Z(\vec{x})=\sum_{\vec{y}\in Y}{\text{e}}^{\left(\sum_{j=1}^{n}\sum_{i=1}^{m}\lambda_{i}f_{i}\left(y_{j-1},y_{j},\vec{x},j\right)\right)}.$$

Here, Y is the set of all possible label sequences over which is summed up, so that a feasible probability is obtained. The factor functions combine different features *f*_*i *_of the considered part of the text and label sequence. We use mainly morphological features of the text tokens for every possible label transition. They have usually a form similar to

$$f_{i}\left(y_{j-1},y_{j},\vec{x},j\right)=\{\begin{array}{ll} 1\text{ if} & y_{j-1}=B\text{-}Hmod\text{ and}\\ & y_{j}=I\text{-}Hmod\text{ and}\\ & x_{j}\text{ starts with a capital}\\ & \text{letter}\\ 0 & \end{array}$$

We use a standard feature set like in [15]. An overview about the used features classes and some examples is depicted in Table 2. Many of the applied features are extracted by standard methods, especially the morphological ones.

**Table 2:** Features applied as parameters of the CRF. Applied features which are used as parameters of the CRF are ordered by their classes, corresponding feature examples and explanations are given.

Name	Explanation
Static morphol. features	Reg.Ex.
All Caps	[A-Z]+
Natural Number	[0–9]+
Alpha-Num	[A-Za-z0–9]+
	
Autom. generated morphol. features	
Autom. Prefixes/Suffixes	Autom. generation of a feature for every token: match that prefix or suffix
	
WordsAsClass	Autom. generation of a feature for every token: match that token
	
Context	
Spaces	Is a token preceded or succeeded by white space
In Brackets	Is a token pre-ceded or succeeded by brackets

Our own implementation of the Named Entity Recognizer of histone modification terms is based on Mallet [28], a widely used and successfully applied system for linear-chain CRF.

To assess the quality of the obtained model the *F*_1 _measure has been calculated which is defined by

$$F_{\beta }=\frac{(1+\beta^{2})\cdot precision\cdot recall}{\beta^{2}\cdot precision+recall}$$

with *β *= 1.

### Corpus generation and annotation

For CRF, a supervised machine learning method, annotated training and testing corpora are required. Two corpora have been annotated for training and testing the CRF.

#### Corpus generation

To train a model an initial corpus (refered to as EPI-TRAIN) of 187 MEDLINE titles and abstracts has been selected manually from a bigger corpus in which both histones and modification terms occur together. This was obtained by a coocurrence MEDLINE search with ProMiner [17] using two separate dictionaries. One contains histone terms and the second one 75 modification terms and spelling variants, like '*di-methylation*' and '*dimethylation*'. With this approach 10,576 articles have been obtained. From that corpus 187 titles and abstracts have been selected manually. It was ensured that every modification type is covered by the corpus. EPI-TRAIN has been annotated with the entity class **Hmod **described below. It comprises 1,605 sentences, 44,876 tokens, and 601 annotated entities. For validation of the trained CRF model and parameter selection a 10-fold cross-validation was performed. For testing the trained model, the corpus EPI-TEST has been generated on the basis of a PubMed search using the MeSH term 'epigenetics'. From 24,653 obtained articles 1,000 titles and abstracts have randomly been chosen and annotated. They are distinct from the articles contained in the EPI-TRAIN corpus. EPI-TEST contains 8,880 sentences, 236,160 tokens, and 221 annotated entities.

#### Corpus annotation

**Figure 2:**
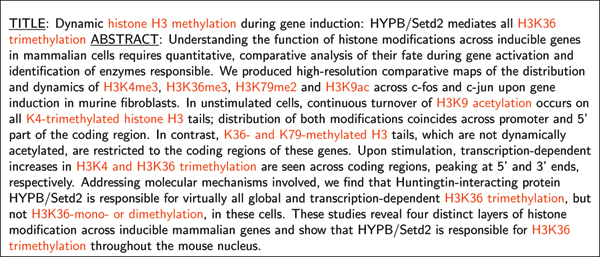
**Annotated example text**. Example title and abstract (PMID:18157086, Edmunds et.al 2008) with histone modifications annotated as entity type **Hmod**.

WordFreak [29] has been used for the annotation of the corpora. The histone modifications occurring in the selected corpora have been annotated as shown in Figure 2. For entity type **Hmod **the term had to contain at least one histone type and one modification term, e.g. '*histone acetylation*' or '*histone 3 dimethylation*'. The removal of a modification, like '*H3K9 demethylation*', has also been annotated, because an existing modification is changed. Instead, if a histone modification is part of an enzyme, e.g. in '*H3K9 methyltransferase*', the term is not annotated. Enumerations are handled as follows: If modification terms, similar to the official nomenclature, occur in an enumeration, like '*H3K36me3, H3K79me3 and H3K9ac*', they have been annotated as single terms. By contrast, long forms, like '*H3K36-mono- or dimethylation*', have been annotated as a whole phrase. The two annotated corpora EPI-TRAIN and EPI-TEST are available in an IOB format from the download webpage [30].

### Standardization and normalization of histone modification term variants

#### Term standardization

The recognition of histone modification descriptions alone is not sufficient. An inevitable next step is to map the different description variants onto standard terms. We developed a procedure for transforming the identified terms into standard terms corresponding to the Brno nomenclature. It includes a set of rules which is applied to every term. First a validity check is performed to filter false positive terms found with the CRF. One basic rule is the absence of a histone type, for instance. If a term passed this filter, additional rules check the histone type, the presence and quality of modifications, the mentioned amino acid, and the position if provided. A translation of '*dimethylated lysine 20 of histone H4*' results in '*H4 K 20 me 2*'. Terms containing enumerations of histone types, modified amino acids, modifications or positions have been resolved by a further set of rules. They define for instance the position dependency between parts of a modification description. From the term '*di- and trimethylation of lysine 4 at histone 3*' two terms '*H3 K 4 me 2*' and '*H3 K 4 me 3*' are generated.

#### Term normalization

The mapping of terms to unique database or ontology identifiers is used e.g. for proteins and genes to annotate further context information to them. We also want to use this way of adding more information to the identified terms. It is what we call normalization in this paper. We investigated following data resources and ontologies for their normalization usability of histone modification standard terms: *Gene Ontology (GO) *[31] (version 1.2 (09-07-2008)), *PSI-Mod *[32], and *HistOn *[33]. The analysis of the resources is given below. Terms from terminologies not corresponding to the standard term form have been transformed by the standardization process described above. Subsequently, they have been used for normalization of the identified terms from text.

### Histone modification hierarchy generation

Scientists working in epigenomic research have different information needs concerning histone modifications. They would like to obtain scientific articles with different focuses for getting an overview of the research in their own or related fields. Some would possibly ask a text retrieval system general questions, like: 'Give me all documents that contain modifications of histone 3'. Others might like to perform a more specific text search, like: 'Give me all documents dealing with trimethylated lysine at position 9 of histone 3'. The first question implicitly includes the second one in this case. It describes a demand that semantic text retrieval systems, like Textpresso [34] and SCAIView [35], can cope with. In such a system the recognized named entities are mapped to concepts of a hierachy which is used for the organization of texts and allows for semantic search.

We analysed the existing hierarchical structured terminologies and ontologies *MeSH *[36], *GO*, *PSI-Mod*, and *HistOn*, for their applicability as histone modification concept hierarchy in such a system. We realized that there is no resource exhaustively covering histone modifications. Therefore, we created an organism-independent hierarchy of standardized histone modification concepts.

In general, the hierarchy could be generated from two different points of view: Modification-centric or histone-centric. We decided for a histone centric organization of the standardized terms, functioning as concepts. Herewith, getting a fast overview about all modification types of a certain histone type is enabled. Furthermore, applied in a semantic text retrieval system, it allows for organizing scientific texts related to one histone type at different granularity levels. A section of the whole generated hierarchy is given below for histone 3 as an example. Five possible methylation states are given (mono-methylation: me 1, di-methylation: me 2, asymmetric di-methylation: me 2a, symmetric di-methylation: me 2s, tri-methylation: me 3, and unspecified modification type: me) at two amino acids (K: lysine and R: arginine) and two positions (2 and 4):

   ...

   0.3.0 H3

   0.3.0.1 H3 me

   0.3.0.1.0.1 H3 R 2 me

   0.3.0.1.0.2 H3 K 4 me

   0.3.0.1.1 H3 me 1

   0.3.0.1.1.1 H3 R 2 me 1

   0.3.0.1.1.2 H3 K 4 me 1

   0.3.0.1.2 H3 me 2

   0.3.0.1.2.1 H3 R 2 me 2

   0.3.0.1.2.2 H3 K 4 me 2

   0.3.0.1.2.a H3 me 2a (asymmetric)

   0.3.0.1.2.a.1 H3 R 2 me 2a

   0.3.0.1.2.s H3 me 2s (symmetric)

   0.3.0.1.2.s.1 H3 R 2 me 2s

   0.3.0.1.3 H3 me 3

   0.3.0.1.3.1 H3 R 2 me 3 

   0.3.0.1.3.2 H3 K 4 me 3

   ...

To every term in the hierarchy a unique number has been assigned. It has at most 7 levels. A basic term set consisting of general histone modification concepts has been assigned to every included histone type. Subsequently, the hierarchy has been populated by standardized terms from *GO*, *MeSH*, *HistOn*, manually collected specific histone modification terms from the antibody online catalogue of *Abcam *[37], and MEDLINE articles. The terms of the developed hierarchy have been automatically compared with the standardized ones from these resources. Those which have not been used so far within the hierarchy have been proposed by the system for its extension. An analysis of the impact of the single term resources is given below. The generated hierarchy is available in an xml format from the download webpage [30] as well.

## Results and discussion

In the following the entity recognition and normalization approach is evaluated and the hierarchy generation is discussed. Additionally, we show the application of the developed approach for epigenomical research.

### Evaluation of the histone modification recognition

The parameter selection has been performed by 10-fold cross-validation. The optimal parameter set has been used to train a model on complete EPI-TRAIN which is applied on the test corpus EPI-TEST.

**Figure 3:**
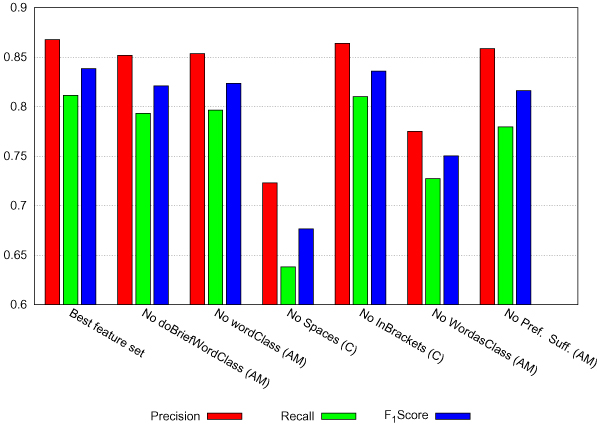
**Results of the feature analysis**. Recall, precision, and *F*_1 _measure are given for every sinlge feature analysis experiment. The non-used features or combinations of them from the two classes *Automatic generated morphological features (AM) *and *Context (C) *are provided.

To prove the impact of features as a parameter of the linear chain CRF, single features or combinations of them have systematically been left out. For every modified feature set a single model has been trained on EPI-TRAIN and was validated by 10-fold cross-validation. The obtained results are shown in Figure 3.

The best feature set has a high performance in recall (0.81), precision (0.87), and *F*_1 _measure (0.84). It includes following features and feature generating methods: *Prefix*, *Suffix*, *InBrackets*, *Words as Class*, *Spaces*, *wordClass*, *and doBriefWordClass*. The features from class *Static morphological *have no impact on the result (data not shown), hence they have been omitted altogether. On the contrary, leaving out *Spaces *and *Words as Class *affect the histone modification term recognition and lead to a considerable decrease in precision, recall, and *F*_1 _measure. The features *Prefix *and *Suffix *have a negative impact onto the recall. It points out that it is relevant whether the token is preceded or succeeded by white space and if words occuring in histone modification descriptions are learned by the system. The first one is important especially in enumerations or abbreviations of terms to separate them from each other. This feature already indicated a high impact on the identification of IUPAC and IUPAC-like names [15].

The optimal feature set was applied to tag the test corpus EPI-TEST. The results for recall, precision, and *F*_1 _measure obtained on EPI-TRAIN and EPI-TEST are provided in Table 3. Compared to the results on the training corpus, tagging of EPI-TEST lead to lower recall (0.76), the same precision (0.87), and a lower *F*_1 _measure (0.81).

**Table 3:** Results of the histone modification recognition approach. Results of the 10-fold cross-validation on the corpus EPI-TRAIN and testing of the model on the independent corpus EPI-TEST. Recall, precision, *F*_1 _measure, and the standard deviation for the cross-validation are given.

	EPI-TRAIN	EPI-TEST
Recall	0.81 (± 0.05)	0.76
Precision	0.87 (± 0.05)	0.87
F1 measure	0.84 (± 0.05)	0.81

### Evaluation of the term standardization and normalization

#### Term standardization evaluation

Entities from the two annotated corpora EPI-TRAIN and EPI-TEST have been used to establish rules for transforming term variants to standard terms according to the nomenclature. Standard terms have been manually assigned to every **Hmod **entity of EPI-TRAIN and EPI-TEST. They have been used for the automatic evaluation of the standardization results. In the first step, rules have been developed using all entities from EPI-TRAIN. Subsequently, they have been tested on entities from EPI-TEST. For reducing the number of false positives, further rules have been incorporated into the system after testing on EPI-TEST entities. The results of the standardization of unique entities from EPI-TRAIN and EPI-TEST given in Table 4 shows a very good performance of the system.

**Table 4:** Results of the term standardization process. Given is the number of annotated histone modification terms (Ann. terms) and the fraction (in %) of correctly standardized terms (Std. terms).

	EPI-TRAIN	EPI-TEST
Ann. terms	414	123
Std. terms	397 (95.89%)	121 (98.37%)

Over 95% of the entities from the EPI-TRAIN corpus and over 98% of the entities from the EPI-TEST corpus have been transformed correctly. Problems occurred for terms like '*H3K9me2S10p*' resulting in '*H3 K 9 S 10 me 2s*' instead of '*H3 K 9 me 2*' and '*H3 S 10 ph*'. Here, the fragmentation and correct assignment of modification states to the amino acids need to be improved.

To test the system on histone modification terms recognized by the CRF, entities tagged on the EPI-TEST corpus have been extracted, standardized and evaluated. The tagged entities have been checked up on false positive terms. They have been divided into four classes. The following list provides a brief description of the classes, some examples, and the fraction of terms contained in the different classes related to all false positives (in %):

1. Modification descriptions without histone mentions (3.7%): '*acetylation and methylation*'

2. Enzymes introducing or removing histone modifications (7.4%): '*H3K9 methyltransferase*'

3. Boundary problems (48%): 'H3 – K9) with no sign of histone H2AX phosphorylation', '*H3K9me3 at pericentric heterochromatin. H3K27me3 and H4K20me*'

4. Terms with other meaning (25%): '*phosphorylation of IRS*', '*eradication of H7N1, H7N3 and H5*'

False positive terms from classes 1, 2, and 4 have been identified by 100% and have been rejected by the system. Instead, false positive terms corresponding to class 3 which contain histone modification mentions have been passed to the standardization process. They have been standardized correctly by 76.5%. Especially, long terms containing many modification types in an enumerations like '*Methylation of H3 at lysine residues K4 and K79 depends on ubiquitylation of histone H2B*' caused errors in the standard term generation. It demonstrates that there is still some room for improvement of the standardization process.

#### Term normalization evaluation

For the normalization of histone modification terms, *Gene Ontology *was considered to be the best resource. It is used as a standard for annotating protein and genes. Unfortunately, it implies only 17 histone modification concepts with different grades of granularity. Only one concept describes a specific histone modification type at a defined protein position. The remaining ones contain general information on histone modifications, like '*histone acetylation*' (GO: 0016573). Furthermore, several modification types are missing, e. g. biotinylation or glycosylation. The analysis result for *HistOn *is similar. It contains 17 histone modification descriptions [33]. The ontology *PSI-Mod *defines general and specific amino acid modification types like 'omega-N, omega-N-dimethyl-L-arginine' which are protein independent. Both are not applicable as normalization resources. *MeSH *provides 13 concepts for histones and one histone modification description. That is why the concepts from *GO *and *MeSH *have been transformed to standard terms and have been used for normalization. The standardizatin of the terms from both resources was 100% correct. The normalization of histone modification terms to *GO *allows for annotating additional information, like genes or proteins, which are linked to the ontology.

### Discussion of the histone modification hierarchy generation

Since there was no existing comprehensive hierarchy ready to use, we developed our own, including 462 concepts. It is a manually created text file which was transformed into xml. The used term resources contribute to the hierarchy concepts as follows:

• 13 histone types: 13 histone types connected to *GO *obtained with Gene product search using AmiGO [38], 13 in *MeSH*, 7 in the online catalogue of Abcam, 8 in *HistOn*, 10 in MEDLINE articles

• 262 general histone modification types: 16 in *GO*, 47 in MEDLINE articles, 1 in *MeSH*

• 156 specific modification types from different resources: 148 from online catalogue of Abcam, 52 in MEDLINE articles, 1 in *GO *and *HistOn*.

The terms from the different resources overlap in content. *GO *and *MeSH *are the best of the considered resources for histone types, whereas Abcam and MEDLINE articles are the most useful resources for general and specific histone modification types. The current version of the created hierarchy covers the most important histone types. With new histone modification findings published in the literature and deposited to the analysed resources, the histone modification concept hierarchy is expected to grow in the future.

### Application of the developed approach for epigenomic research

The CRF model trained with the best feature set was applied for tagging the complete MEDLINE. The obtained article number for the most often occurring histone modification types is provided in Table 5.

**Table 5:** Application of the developed approach: number of Medline articles for the most often occuring histone modifications. Number of obtained MEDLINE abstracts for the most often occurring histone modifications after term identification and standardization. (The histone modification example '*H3K9me3*' introduced in Section Background is marked in bold.)

Modification type	Number of articles
H3 K 9 me	231
H3 K 4 me	173
H3 K 4 me 3	104
**H3 K 9 me 3**	**90**
H3 K 9 me 2	80
H3 S 10 ph	79
H3 K 27 me 3	71
H3 K 9 ac	62
H3 K 27 me	60
H3 K 4 me 2	58

It demonstrates that the developed approach is able to link a huge number of abstracts from MEDLINE (version of the 23th June 2008) to one histone modification concept at once. It shows the importance of the mapping of the different term variants onto one standard concept. In comparison, a simple search strategy implies that all term variants need to be given one by one to the query machinary to find all related texts (cf. PubMed search results in Section Background).

The integration of the histone modification concept hierarchy into the semantic text retrieval and analysis system SCAIView and the mapping of the hierarchy concepts to the recognized ones enables the retrieval of articles containing histone modifications at different levels of granularity. With SCAIView, which was developed in our group, a combined search with various other biomedical concepts, like proteins, genes, diseases, and chemical substances/drugs can be performed supporting an easy navigation through huge text corpora, like MEDLINE. These entities are detected by our dictionary-based Named Entity Recognition (NER) tool ProMiner [17] and two other CRF-based NER approaches which are optmimized for the identification of SNPs [23] and IUPAC-like names [15]. Having this, further studies can be performed by a combined analysis of additional biomedical concepts relevant for epigenomic research. This could lead to new hypotheses directing the design of further experiments.

## Conclusion

We have introduced an approach for the automated identification of histone modification mentions in text with CRFs which reaches high *F*_1 _measures on training (0.84) and test corpus (0.81). The standardization of the identified terms, which has a very good performance of 95–98%, enables the mapping of different spelling variants of one histone modification type onto each other. We showed that our new developed approach is superior to a PubMed search for retrieving a high number of abstracts related to a histone modification type in MEDLINE. The integration of the developed histone modification hierarchy into a semantic text retrieval system and its mapping to standardized terms enables semantic search. The combination of a search for other biomedical named entities allows for asking more complex questions in one single step, which has not been possible up to now. Furthermore, thanks to the normalization of histone modification terms to *GO *and *MeSH *additional epigenomical relevant information, like influenced genes or proteins can be annotated.

Future work has to be invested in the extraction of histone modification related information. Finding related expression states of certain genes, DNA methylation states, cell/tissue types, chemical substances, phenotypes, and disease states for example, will improve literature-based knowledge discovery and thus support hypothesis generation for epigenomics.

## Competing interests

The authors declare that they have no competing interests.

## Authors' contributions

CK selected and annotated the corpora, developed the term standardization and term normalization process, generated the hierarchy, performed the CRF studies and analyzed the features, as well as evaluated the data and drafted the manuscript. RK implemented the workflow from annotating and tokenizing text to training, tagging and evaluation of Conditional Random Fields. He was involved in the parameter selection of the models. MH-A critically revised the manuscript.
